# Higher alcohol use is associated with subsequent increased risk seeking toward gains: A longitudinal cohort study in young men

**DOI:** 10.1111/acer.70051

**Published:** 2025-04-19

**Authors:** Angela Hentschel, Johannes Petzold, Hao Chen, Andreas Heinz, Michael N. Smolka

**Affiliations:** ^1^ Department of Psychiatry and Psychotherapy Technische Universität Dresden Dresden Germany; ^2^ Department of Psychiatry and Psychotherapy Charité – Universitätsmedizin Berlin, Campus Charité Mitte Berlin Germany

**Keywords:** alcohol use, decision making, delay discounting, loss aversion, risk taking

## Abstract

**Background:**

A higher propensity for impulsive and risky choices has often been reported in individuals with addiction, such as alcohol use disorder (AUD). Although domains of choice impulsivity, for example, temporal discounting, have been identified to predispose the development of hazardous alcohol use, research on altered decision making as a consequence of drinking is scarce. These may be particularly pronounced during periods of progressive brain development, such as young adulthood.

**Methods:**

This 3‐year prospective study investigated the effects of alcohol use on changes in four decision‐making domains in 18‐year‐old men (*N* = 130). We assessed temporal changes in discounting of delayed rewards, risk aversion for gains, risk seeking for losses, and loss aversion. By correlating three‐year cumulative alcohol consumption and total binge drinking frequency with respective change scores, we aimed to explore the influences of drinking on altered development in different impulsive choice tendencies.

**Results:**

From ages 18 to 21, choice impulsivity in our moderately drinking cohort decreased significantly with respect to temporal discounting and risk aversion for gains, while risk seeking for losses and loss aversion did not change significantly. Importantly, higher cumulative alcohol intake and more binge drinking occasions over 3 years were associated with slower increases in risk aversion for gains, that is, the general trend for lower choice impulsivity was diminished. Such relationships were not found for temporal discounting, risk seeking for losses, or loss aversion.

**Conclusions:**

Alcohol consumption in young adulthood is linked to stunted development of risk aversion for gains. Given that risk seeking for gains was previously identified as a risk factor for increasing alcohol intake, this relationship may reinforce a spiral of escalating consumption over time. The absence of similar findings in other decision‐making domains suggests that drinking behavior and modifying factors interact differently across domains, rather than universally enhancing impulsive choice behavior.

## Graphical abstract

In moderate‐drinking males, from ages 18 to 21, we found decreasing temporal discounting and increasing risk aversion for gains. Higher cumulative alcohol intake and more frequent binge drinking were linked to a stunted development in risk aversion for gains. Since risk seeking for gains is a known risk factor for increasing alcohol use, our findings suggest a bi‐directional relationship that may fuel escalating consumption over time. Future research should explore the underlying neurofunctional mechanisms and identify environmental modifiers of this process.

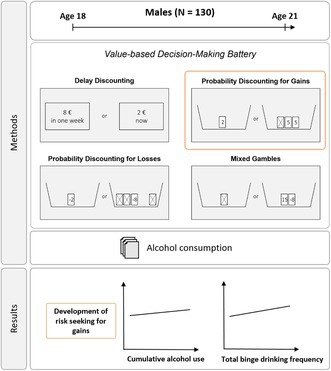

## INTRODUCTION

Impulsive decision making, as one domain of impulsivity (Castellanos‐Ryan et al., 2014), has been suggested to play an important role in drug‐seeking behavior and the manifestation and maintenance of addictive disorders, such as alcohol use disorder (AUD) (e.g., Heinz et al., 2011). A common feature marking the transition from occasional substance use toward addiction is that individuals consume the drug repeatedly in large quantities, despite being aware of negative future consequences on health and psychosocial functioning (American Psychiatric Association, 2013). Thus, the repeated choice for a misused substance by individuals facing such negative consequences may involve choice impulsivity and impairments in cognitive control (Poulton & Hester, 2019), which in turn have been linked to early adversity and social exclusion stress (Brandt et al., 2022). Indeed, alterations within different facets of impulsive choice, which seem to favor drug use, have been linked repeatedly to the misuse of various drugs (e.g., MacKillop et al., 2011). However, it is important to clarify not only whether aberrant decision making represents predisposing factors for alcohol misuse, but also how alcohol use causes alterations in brain functioning that impact impulsive choice behavior.

Choice impulsivity comprises different domains of value‐based decision making (VBDM) that involve delayed or uncertain rewards and losses. They are typically assessed using questionnaires or tasks that provide forced choices for one of two offers. One domain of VBDM entails reward delay impulsivity, which is defined by an individual's tendency to prefer smaller immediate over larger delayed rewards (Swann et al., 2002). This phenomenon can be quantified using temporal or *delay discounting (DD)* tasks. Although, DD is usually assessed using monetary gains (e.g., “Take €5 now or €10 in 2 weeks?”), it is applicable to other reinforcers, such as misused substances, where individuals prefer the immediate drug effect over long‐term health benefits. DD describes the degree *k* to which an individual tends to diminish the subjective value *V* of a reinforcer *A* hyperbolically with increasing reward delay *D* (Mazur, 1987):

$$V=\frac{A}{1+k*D}.$$

Higher values of *k* mean that the individual discounts delayed rewards more steeply, which is considered more impulsive and reflects a preference for immediate gratification (e.g., Green et al., 1994). Steeper DD was consistently linked to AUD (e.g., Bernhardt et al., 2017; MacKillop et al., 2011) and its early onset (Dom et al., 2006; Kollins, 2003), independent of comorbid psychopathology (Gowin et al., 2019). Apart from AUD, steeper discounting was also related to heavy social and problem drinking (Vuchinich & Simpson, 1998).

Another facet of decision making involves risk taking for rewards or losses. A meta‐analysis by Chen et al. (2020) reported that heavy chronic or dependent alcohol users showed an increased propensity for risky choices compared with non‐ or light users, which was associated with the time in treatment. Risk aversion is defined by an individual's tendency to favor smaller certain over larger probabilistic rewards or losses, whereas risk seeking tends to favor the higher, probabilistic options. For both gains and losses, *Probabilistic Discounting (PD)* describes the devaluation of the subjective value *V* of a risky amount *A* (gain or loss) with increasing uncertainty to receive it. The probability *p* to receive the risky amount is transformed to the odds *against* the event of winning or losing: $ϴ=\left(1-p\right)/p$. The decrease of the subjective value follows a hyperbolic function (Rachlin et al., 1991):

$$V=\frac{A}{1+k*ϴ}.$$

Similar to DD, higher values of *k* represent higher rates of discounting. In the case of rewards, higher PD reflects risk *aversion*, since the perceived value of the probabilistic gain declines more steeply with greater uncertainty to receive it. In the case of losses, higher PD reflects risk *seeking*, since likewise the absolute value of the probabilistic loss is devalued more steeply. Studies have linked AUD and heavy drinking to risk‐seeking behavior for gains (e.g., Bernhardt et al., 2017; Brevers et al., 2014; Takahashi et al., 2009). Regarding PD for losses, mixed findings have been reported. While one study observed risk aversion in people with AUD compared with healthy controls (Bernhardt et al., 2017), another study found no differences between these groups (Brevers et al., 2014). Such relationships regarding PD for gains or losses were not found toward the frequency of use in mildly drinking individuals (Takahashi et al., 2009).

When risky choices involve both gains and losses, individuals are usually more sensitive toward losses and thus reject a 50/50 gamble if possible gains are not substantially larger than possible losses (Tom et al., 2007). This phenomenon is termed *loss aversion* (Kahneman & Tversky, 1979), whose degree *λ* linearly decreases the subjective value *V* of a 50/50 gamble between a gain *G* and a loss *L* (Frydman et al., 2011; Pooseh et al., 2018):

$$V=\frac{1}{2}\;\left(G-\mathrm{\lambda L}\right).$$

The decision whether to accept the gamble is defined by the ratio of the gain to the loss, in which the loss is weighed by *λ*. Higher values of *λ* mean that the individual is more averse to losses and driven to reject the gamble. AUD was associated with low loss aversion, resulting in an increased propensity for high‐risk choices (e.g., Bernhardt et al., 2017; Brevers et al., 2014).

The discussed cross‐sectional studies demonstrate how impulsive decision making is linked to heavy drinking and AUD; however, they cannot distinguish whether these tendencies represent predisposing factors or whether they are themselves altered along with heavy alcohol use, either as a direct consequence or by means of third factors, such as environmental stressors. Longitudinal studies are essential to discern the directionality of these relationships. Disruptive effects of drinking may be more pronounced, especially during stages of ongoing brain development (Jacobus & Tapert, 2013). This includes adolescence but also young adulthood, as brain maturation continues prominently into the 20s (Bethlehem et al., 2022; O'Rourke et al., 2020). Two 2‐year longitudinal studies of adolescents aged 12–14 years at study enrollment employed cross‐lagged designs to examine whether DD predicts subsequent alcohol involvement or vice versa. They found that DD predicted higher alcohol involvement after 6 and 12 months, respectively, but reversely alcohol use did not predict subsequent DD (Fernández‐Artamendi et al., 2018; Fernie et al., 2013). Another longitudinal study showed that higher DD at age 14 predicted an increase in alcohol use over the following 8 years, but cumulative consumption from ages 14 to 22 did not correlate with DD trajectories (Fröhner et al., 2022). In contrast, a study that followed 14‐year‐olds annually over 5 years reported a positive, bi‐directional relationship between drinking behavior and DD from the past to the following survey year (Do & Shin, 2017). In the risk domain, a study assessing 12‐ to 13‐year‐olds every 6 months over 2 years found that higher risk taking predicted subsequent alcohol use, but not vice versa (Fernie et al., 2013). However, these findings emerged in very young samples with very low consumption rates. For example, at baseline 52% had never consumed any alcohol in one study (Fröhner et al., 2022), and another reported an average baseline frequency of only a few drinking episodes per year (Fernie et al., 2013). Such low drinking rates, combined with the reliance on mostly cross‐lagged designs using relatively short predictive windows (6–12 months), do not allow conclusions about long‐term consequences of alcohol use, which may develop after prolonged exposure to higher amounts of alcohol consumption. Alcohol use during adolescence and young adulthood has generally been associated with increases in impulsivity and sensation seeking (Quinn et al., 2011; White et al., 2011), as well as impairments in reward processing and anticipation, predicting hazardous consumption and alcohol‐related problems later on (e.g., Boecker‐Schlier et al., 2017; Cservenka et al., 2015). However, associations between long‐term alcohol use and decision‐making development beyond DD remain poorly studied.

This prospective study sought to fill this gap by investigating the consequences of alcohol use during early adulthood on changes in different tendencies of impulsive choice over 3 years. In a sample of young men, we assessed alcohol consumption quantity and binge drinking frequency along with four domains of decision making: DD, probability discounting for gains (PDG), probability discounting for losses (PDL) and mixed gambles (MG, i.e., loss aversion). The key question was whether long‐term alcohol use contributes to differences in decision making reported between AUD and healthy populations. Thus, based on these differences, we hypothesized that higher levels of cumulative alcohol use and total binge drinking frequency throughout the study would be associated with altered levels of decision making after 3 years: (1) steeper DD; (2) shallower discounting of probabilistic gains (i.e., less risk averse to gains); (3) shallower discounting of probabilistic losses (i.e., less risk seeking to losses); and (4) lower loss aversion. This study is a follow‐up to Bernhardt et al. (2017) and Petzold et al. (2023), who studied the sample in terms of how VBDM at age 18 cross‐sectionally and longitudinally predicted various indicators of alcohol use and dependence.

## MATERIALS AND METHODS

### Participants and procedure

The collection of participant data was part of a bicentric longitudinal study in a community sample of young social drinkers from the German cities of Dresden and Berlin, which aimed to investigate the role of learning and related brain activity in the development of AUD (LeAD Study, ClinicalTrial.gov Identifier: NCT01744834). Local registration offices randomly selected 1937 18‐year‐old men to participate in the study. To avoid potential confounds from sex differences, only men were recruited, given documented disparities in decision making (e.g., Bos et al., 2013) and neurocognitive functioning (e.g., Squeglia et al., 2009). Exclusion criteria were a history of major neurological or psychiatric disorders according to DSM‐IV (except tobacco or mild alcohol dependence), any contraindications to magnetic resonance imaging (MRI), left‐handedness, and fewer than two drinking occasions in the past 3 months. Of 201 participants who completed the baseline assessment, three were excluded due to violations of the participation criteria. This yielded a baseline sample of 198 men at age 18. As part of the LeAD consortium, participants were enrolled between 2012 and 2015 and invited for follow‐up assessments over 6 years. However, the experiments relevant to this study were conducted only in the first 3 years (last assessment 2018), so data from ages 18 to 21 are analyzed here. Sample data were reported in previous studies (Bernhardt et al., 2017; Deza Araujo et al., 2018; Petzold et al., 2023). Interviews and self‐report questionnaires were used to assess alcohol consumption every 6 or 12 months. To capture different aspects of impulsive choice behavior and how they develop over time, the computerized VBDM task battery was used at baseline and after 3 years. Furthermore, cognitive abilities were assessed including working memory, crystallized intelligence, and processing speed. Of the baseline sample, 130 participants completed at least one of the VBDM tasks at the 3‐year follow‐up and were therefore included for the analysis. Sample characteristics are shown in Table 1. This study was approved by the ethics committees of the Charité Universitätsmedizin Berlin and Technische Universität Dresden. All participants gave written informed consent prior to participation and received monetary compensation.

**TABLE 1:** Descriptive statistics of demographics, value‐based decision making, and alcohol consumption.

	Assessment at month						
	0	6	12	18	24	30	36
Age	18.4 ± 0.2^a^ *N* = 130	19.0 ± 0.3^a^ *N* = 113	19.4 ± 0.2^a^ *N* = 123	20.0 ± 0.2^a^ *N* = 96	20.4 ± 0.2^a^ *N* = 120	20.9 ± 0.2^a^ *N* = 93	21.5 ± 0.3^a^ *N* = 129
Education in years	11.6 ± 0.9^a^ *N* = 130						14.2 ± 0.8 *N* = 122
Value‐based decision making							
DD log(*k*)	−4.60 ± 2.15^a^ *N* = 126						−5.20 ± 2.75 *N* = 129
PDG log(*k*)	−0.20 ± 0.82^a^ *N* = 127						−0.03 ± 1.01^a^ *N* = 127
PDL log(*k*)	−0.26 ± 1.14^a^ *N* = 128						−0.08 ± 1.04^a^ *N* = 127
MG log(*λ*)	0.30 ± 0.70 *N* = 124						0.27 ± 0.74 *N* = 126
Choice consistency log(*β*)^b^	−0.003 ± 0.97^a^ *N* = 119						−0.002 ± 0.97 *N* = 125
Alcohol consumption							
Age of first drink	14.3 ± 1.5^a^ *N* = 130						
Age of first binging (at least 5 drinks)	16.4 ± 0.8^a^ *N* = 89						
Binge drinking frequency during past year			3.0 ± 10^a^, ^b^ *N* = 115		4.0 ± 10^a^, ^b^ *N* = 95		1.0 ± 5^a^, ^b^ *N* = 128
Average intake during past year (g/day)	8.6 ± 16.3^a^, ^b^ *N* = 130		8.5 ± 21.6^a^, ^b^ *N* = 115		8.6 ± 15.9^a^, ^b^ *N* = 94		8.6 ± 16.5^a^, ^b^ *N* = 129
AUDIT‐C		4.0 ± 2.0^a^, ^b^ *N* = 69	4.0 ± 3.5^a^, ^b^ *N* = 86	4.0 ± 3.0^a^, ^b^ *N* = 94	4.0 ± 3.0^a^, ^b^ *N* = 97	4.0 ± 3.0^a^, ^b^ *N* = 102	4.0 ± 3.0^a^, ^b^ *N* = 129

*Note*: Values represent the mean (^c^median) ± standard deviation (^c^IQR) and the sample size (*N*) of non‐missing data.

Abbreviations: DD, delay discounting; MG, mixed gambles (loss aversion); PDG, probability discounting for gains; PDL, probability discounting for losses.

^a^ Measure violates normal distribution according to Shapiro–Wilk test (*p* < 0.05).

^b^ Average of log‐transformed and mean‐centered beta values of DD, PDG, PDL, and MG.

### Measures of alcohol use

Alcohol consumption was assessed with the Munich Composite International Diagnostic Interview (M‐CIDI; Jacobi et al., 2013; Wittchen & Pfister, 1997) and the Alcohol Use Disorders Identification Test (AUDIT; World Health Organization et al., 2001). The CIDI was conducted each year as an in‐person assessment at baseline and after 36 months and via phone after 12 and 24 months. It comprises quantity‐frequency measures of past‐year consumption. This includes the number of standard drinks (SD) per drinking occasion, which were converted to grams of alcohol (12 g per SD, Kuitunen‐Paul et al., 2017), and a categorical measure of drinking frequency, from which the number of drinking occasions was estimated (abstinent = 0 days, less than once a month = 0.25 days/week, 1–3 days a month = 0.5 days/week, 1–2 days per week = 1.5 days/week, 3–4 days a week = 3.5 days/week, (almost) daily = 7 days/week). The amount (gram) of alcohol per occasion was multiplied by the weekly number of drinking occasions and converted into daily consumption levels to gain the average daily intake during the past year. Furthermore, the CIDI assessed the number of past‐year binge drinking occasions, where at least 5 drinks per occasion were consumed. The AUDIT was collected as a self‐report every 6 months online or via mail, beginning 6 months after the baseline (i.e., no AUDIT at baseline). The AUDIT‐C was extracted to gain a pure consumption score. It sums up the first three items of the AUDIT, including categorical measures of drinking frequency, the number of drinks per occasion, and binge drinking frequency as defined by 6 or more alcoholic beverages per day.

To gain estimates of the total consumption over 3 years, the average daily intake for the past year was converted into total kilograms of alcohol per year (365 days). The past‐year amount and binge drinking frequency were each summed up for the 12, 24, and 36‐month follow‐up to gain the total consumption in kg and the total number of binge drinking occasions, respectively, for the 3‐year period. Missing values of individual drinking scores per time point were imputed using the individual's mean score.

### Value‐based decision‐making battery

**FIGURE 1:**
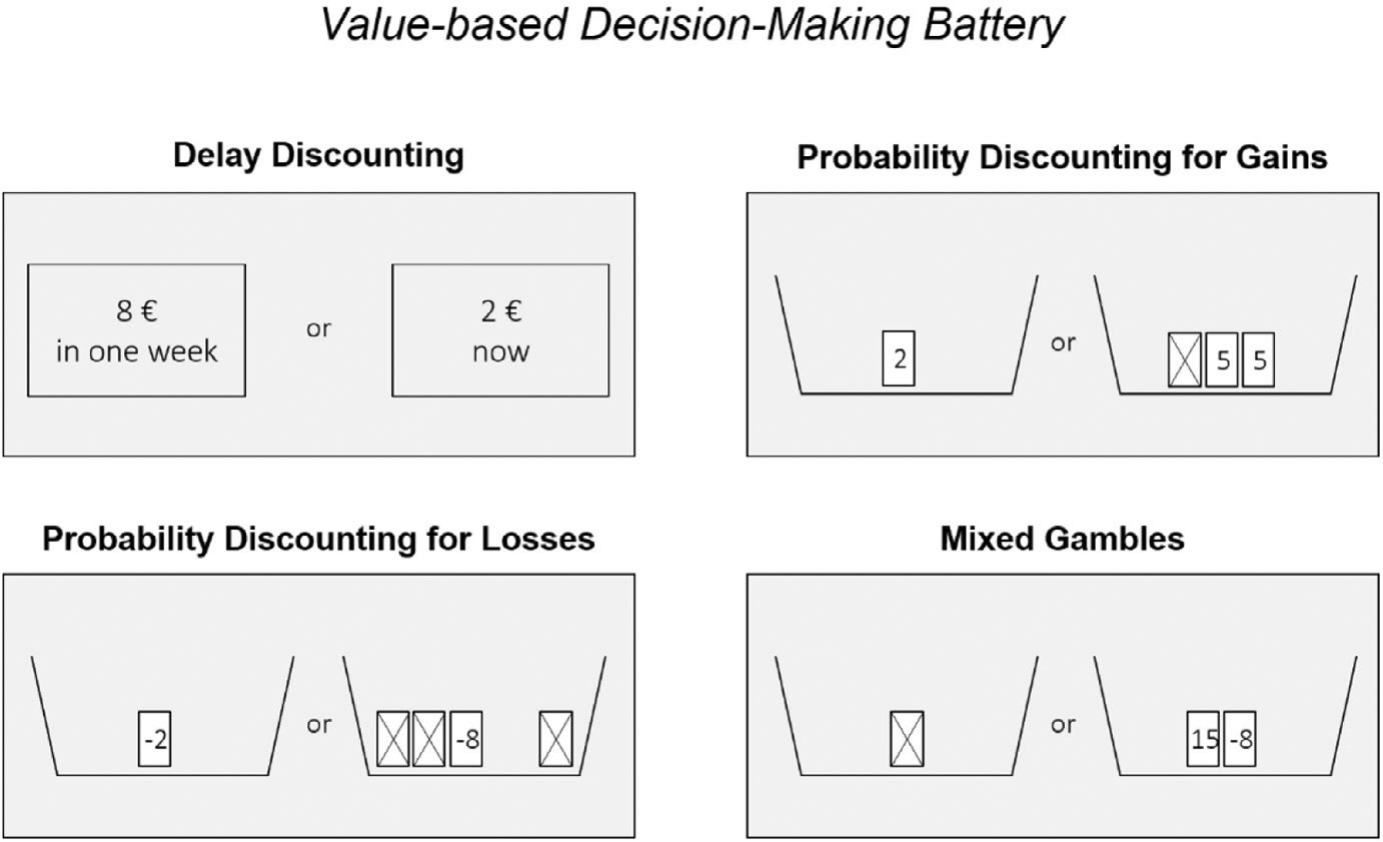
Value‐based decision‐making (VBDM) battery comprised four tasks: delay discounting (DD), probability discounting for gains (PDG), probability discounting for losses (PDL), and mixed gambles (MG); for details, see Pooseh et al. (2018). Every task included forced choice trials for one of two offers. Within the DD task, a smaller immediate versus a higher delayed reward was offered. The PD tasks offered a certain smaller versus a probabilistic larger gain (PDG) or loss (PDL). The MG task incorporated rejecting or accepting a 50/50 gamble between a monetary gain versus loss.

The VBDM battery comprised four separate tasks, each capturing a different aspect of impulsive and risky choice behavior: DD, PDG, PDL, and loss aversion assessed via MG. Every trial of each task involved a forced choice for one of two offers of a monetary gain or loss (Figure 1). In the DD task, participants decided between a smaller immediate versus a larger delayed reward, with delays set to 3, 7, 14, 31, 61, 180, or 365 days and rewards ranging from 0.30 to €10. In the PD tasks, choices were between a certain smaller and a probabilistic larger monetary gain (PDG) or loss (PDL). Probabilities for both tasks were set to ⅔, ½, ⅓, ¼, or ⅕, and values ranged from 0.30 to €10 for PDG and −0.30 to €−10 for PDL. In the MG task, participants received a €10 credit before accepting or rejecting a 50/50 gamble between a gain (1 to €40) versus loss (−5 to €−20). The number of trials was 30 for the DD, PDG, and PDL tasks, and 40 for the MG task, without time limit or outcome feedback. Both options were presented simultaneously, with one on the left and the other on the right side of the screen, randomly switching throughout the trials. Participants were instructed that one trial per task would be randomly selected and added to their compensation at the end of the experiment. The battery was presented using MATLAB Release 2010a (The MathWorks, Inc., Natick, MA, United States) and Psychtoolbox 3.0.10 based on the Psychophysics Toolbox extensions (Brainard, 1997; Pelli, 1997).

Individual parameters of DD, PDG, PDL (*k*) and loss aversion (*λ*) were estimated on a trial‐by‐trial basis using a Bayesian adaptive approach. Within this framework, the choice in each trial updates the estimate of *k*/*λ* from the previous trial. At the same time, the offer for the next trial is adapted toward the individual's indifference point of the parameters, at which either of two options is equally likely to be chosen, rendering the choice most informative to infer the underlying true parameter value. The likelihood of choosing one of the two options is modeled by a softmax probability function, in which an inverse temperature parameter *β* describes the consistency of choices. High *β*‐values represent more consistent choices, in which the difference in offered values has great influence on the probability of choosing the subjectively more valuable option. For details on the mathematical framework, please refer to Pooseh et al. (2018). There were single missing data of individual final estimates due to technical issues, which renders their missing pattern completely at random and thereby negligible. Log‐transformation was used to yield symmetrical distributions of the parameters.

### Data analysis

All analyses were performed using R Statistical Software (v4.0.3, R Core Team 2020, Vienna, Austria, https://www.R‐project.org/). Since most of the variables were not normally distributed according to the Shapiro–Wilk test (*p* < 0.05) and to account for extreme outliers, Spearman's correlation coefficients (*ρ*) and paired Wilcoxon tests (two‐tailed) were used to examine correlations and differences between repeated measures. In case of the MG variable which conformed to normal distribution at both time points, results of Pearson's correlation and paired *t*‐test did not substantially differ from the non‐parametric counterparts. The consistency of choices was obtained by averaging mean‐centered *β*‐values from the four VBDM domains. Change scores of VBDM and choice consistency were calculated by subtracting the estimates at age 18 from those at age 21. The change scores were correlated with the cumulative amount of drinking and the total number of binge drinking occasions during the same 3‐year period to investigate effects of alcohol use on changes across different domains of decision making. Confidence intervals of the correlation coefficients were calculated via z‐transformation. One concern in longitudinal studies is potential sample bias due to individual dropouts from the study, especially if certain groups, such as highly impulsive individuals, are underrepresented. We therefore compared baseline values of VBDM between the study sample (*N* = 130) and individuals who did not complete the VBDM follow‐up (*N* = 68), using an unpaired Wilcoxon test, to explore whether choice impulsivity of the dropout individuals substantially differed from individuals who completed the follow‐up.

## RESULTS

### Alcohol use

**FIGURE 2:**
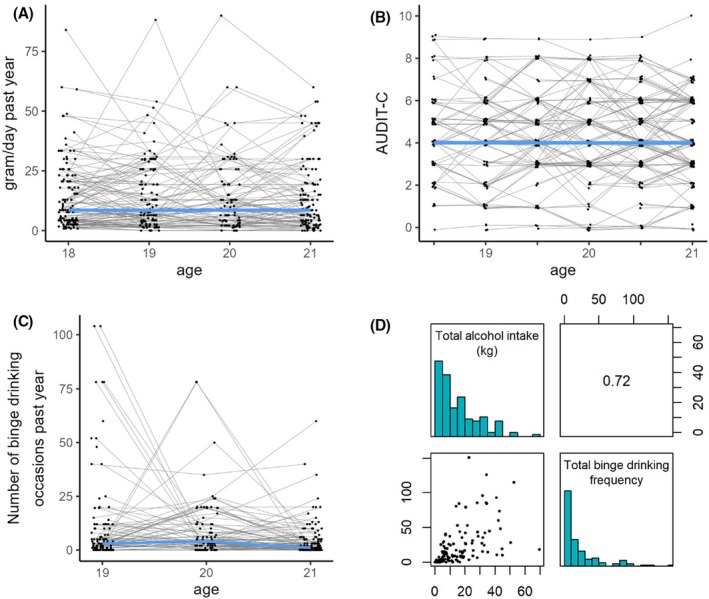
Alcohol use from age 18 until age 21. (A–C) Individual drinking trajectories. Blue lines refer to the median. (D) Distribution of cumulative consumption, which represents the sum of all assessments over 3 years, excluding the year before baseline. Number at the top right refers to Spearman's correlation.

Median levels of average alcohol intake remained moderate throughout the study (compare Figure 2 and Table 1), with 8.6 g/day (IQR = 16.3) the year before baseline and 8.6 g/day (IQR = 16.5) the year before the 36‐month follow‐up (FU36). Correlating the individual's daily intake with the months of assessments revealed no linear change in consumption over time at the group level (*ρ* = −0.04, *p* = 0.397). The median trajectory of AUDIT‐C scores showed a similarly stable pattern (*ρ* = −0.01, *p* = 0.768), with 4.0 (IQR = 2.0) at FU06 and 4.0 (IQR = 3.0) at FU36. Median numbers of binge drinking events during the past year remained very low and declined slightly over time (*ρ* = −0.16, *p* = 0.004), with 3.0 (IQR = 10.0) binge drinking events throughout the year before FU12 and 1.0 (IQR = 5.0) event the year before FU36. The highest levels of alcohol consumption were 90 g/day and 104 binge drinking events throughout the year. Over 3 years, young men on average consumed 11.5 kg (IQR = 15.6) of alcohol and had 10.0 (IQR = 25.0) binge drinking events, classifying most of the sample as moderate daily drinkers and low‐frequency binge drinkers. Carr et al. (2024) reported that an average intake of at least 20 g/day triples the risk of developing an AUD, equating to a total of 21.9 kg of alcohol over 3 years. In our sample, 24% of the young men consumed alcohol above this level. Both measures of cumulative consumption (total kg and total number of binge drinking occasions) were strongly correlated (*ρ* = 0.72, Figure 2D).

### Development in VBDM and cognitive abilities during early adulthood

**FIGURE 3:**
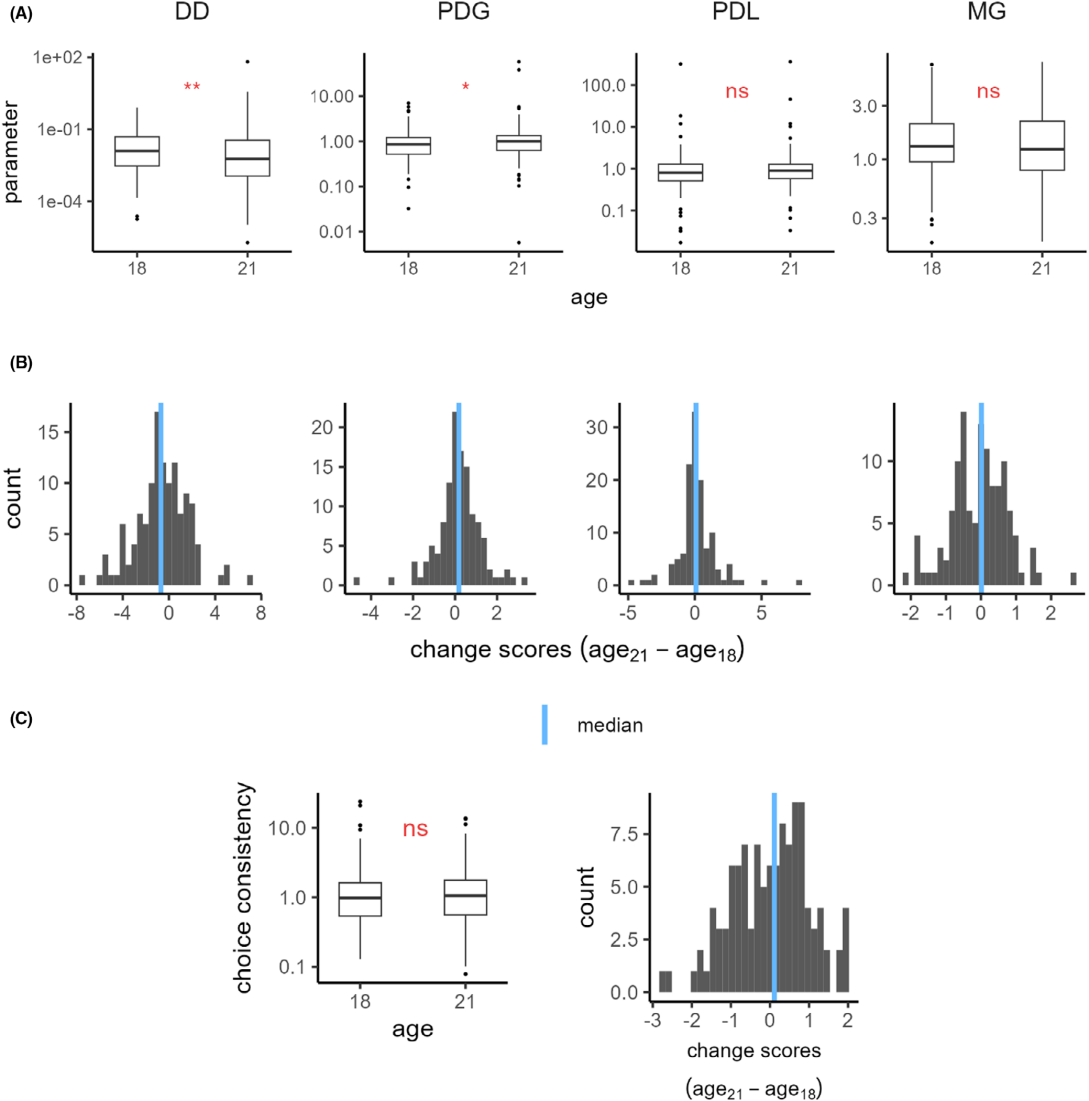
Development in different domains of value‐based decision making from ages 18 to 21. (A) Boxplots of raw parameter estimates and (C, left) choice consistency at both ages. Only individuals with non‐missing values are included. (B) Distribution of change scores of the parameters and (C, right) choice consistency, calculated by subtracting log‐transformed values at age 18 from those at age 21. Blue lines refer to the median. DD, delay discounting; MG, mixed gambles (loss aversion); PDG, probability discounting for gains; PDL, probability discounting for losses. Asterisks refer to the Wilcoxon signed‐rank test for paired samples: **p < 0.01, *p < 0.05.

When examining changes in VBDM over time, comparing estimates at ages 18 and 21 revealed decreasing preferences for immediate monetary rewards (DD, *W* = 2754, Md_18_ = −4.38, Md_21_ = −5.15, *p* = 0.004) and increasing risk aversion to gains (PDG, *W* = 4856, Md_18_ = −0.15, Md_21_ = 0.0007, *p* = 0.014) (compare Table 2 and Figure 3). Given that estimates were transformed using natural logarithms, this represents substantial rates of change from the baseline, with a 50% decrease in DD and a 20% increase in PDG at the group level. No significant change was found in risk seeking toward losses (PDL, *W* = 4437, Md_18_ = −0.22, Md_21_ = −0.10, *p* = 0.219) or loss aversion (MG, *W* = 3434, Md_18_ = 0.27, Md_21_ = 0.21, *p* = 0.609). Furthermore, no significant change over time was found in choice consistency (*W* = 3395, Md_18_ = −0.02, Md_21_ = 0.05, *p* = 0.741). Addressing data quality and potential sample bias in estimates of VBDM due to participant dropouts, comparisons of VBDM at age 18 between the baseline sample and the dropout group revealed no significant differences for any VBDM task, as illustrated in Figure S2. Regarding changes in cognitive domains, the young men improved significantly from age 18 until age 21 in all tested domains, including working memory, crystallized intelligence, and processing speed (for details, please refer to the Table S1).

**TABLE 2:** Development in value‐based decision‐making (VBDM) domains and choice consistency between age 18 and age 21.

VBDM	Age	Median	Diff (Age_21_–Age_18_)	Wilcoxon signed‐rank test			
				*W* test statistic	(pseudo) median [CI_95_]	*p*‐value	*N*
DD log(*k*)	18	−4.38	−0.76	2754	−0.58 [−0.97, −0.19]	0.004**	125
	21	−5.15					
PDG log(*k*)	18	−0.15	0.16	4856	0.19 [0.04, 0.35]	0.014*	124
	21	0.0007					
PDL log(*k*)	18	−0.22	0.12	4437	0.09 [−0.06, 0.26]	0.219	125
	21	−0.10					
MG log(*λ*)	18	0.27	−0.06	3434	−0.04 [−0.18, 0.10]	0.609	120
	21	0.21					
Choice consistency log(*β*)^a^	18	−0.02	0.07	3395	0.03 [−0.16, 0.23]	0.741	114
	21	0.05					

*Note*: Differences between both ages were tested using Wilcoxon signed‐rank tests for paired samples.

Abbreviations: CI_95_, 95% confidence interval; DD, delay discounting; MG, mixed gambles (loss aversion); PDG, probability discounting for gains; PDL, probability discounting for losses.

^a^ Average of log‐transformed and mean‐centered beta values of DD, PDG, PDL, and MG.

Asterisks refer to the Wilcoxon signed‐rank test for paired samples: **p < 0.01, *p < 0.05.

### Effect of alcohol use on VBDM

**FIGURE 4:**
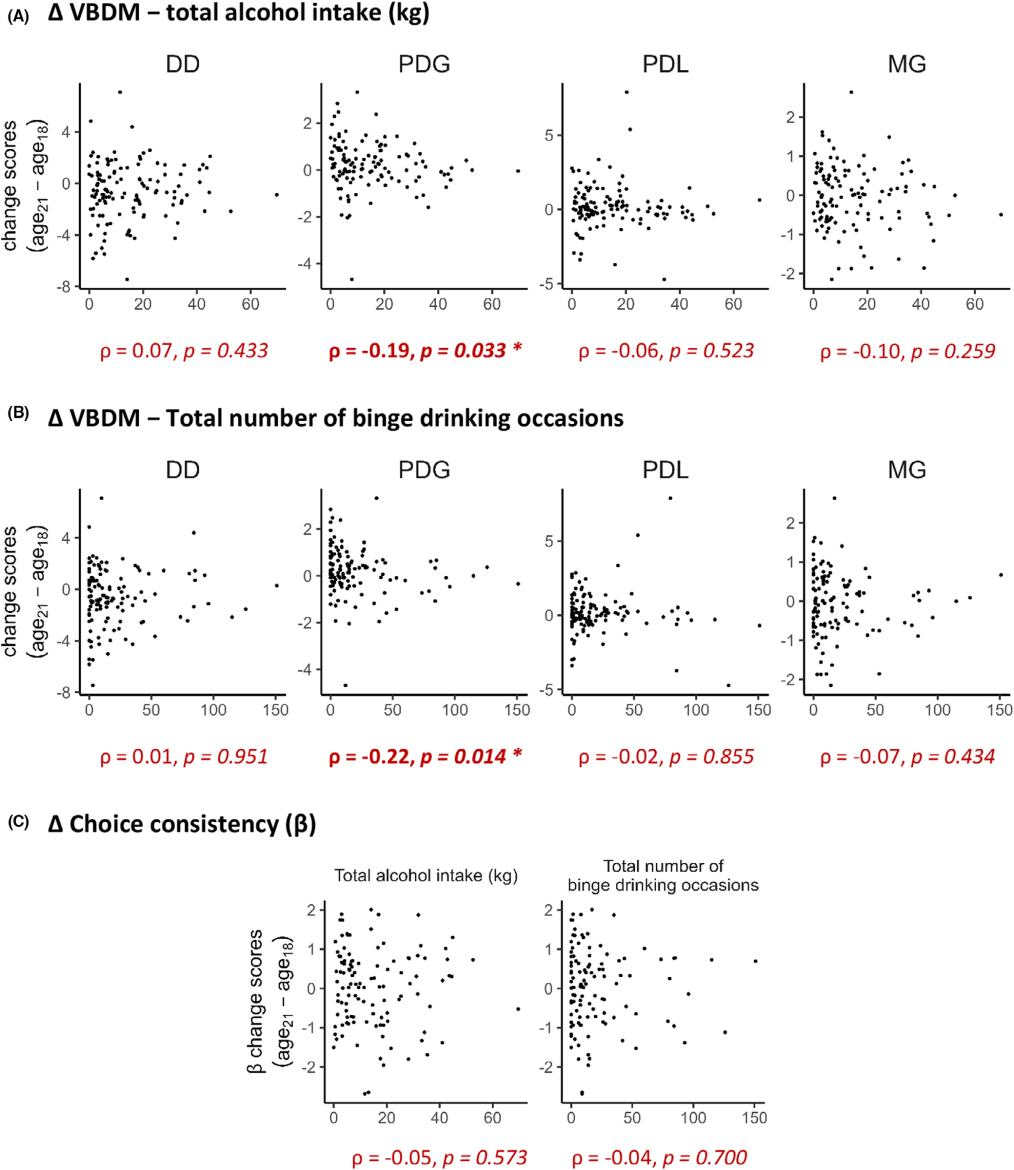
Associations between cumulative alcohol consumption and change scores of (A, B) different decision‐making dimensions as well as (C) choice consistency from ages 18 to 21. Change scores were calculated by subtracting log‐transformed values at age 18 from those at age 21. Values of choice consistency represent the average of log‐transformed and mean‐centered *β*‐values of DD, PDG, PDL, and MG. Spearman correlations (*ρ*) are shown at the bottom. DD, delay discounting; MG, mixed gambles (loss aversion); PDG, probability discounting for gains; PDL, probability discounting for losses.

To test whether alcohol use from ages 18 to 21 alters development in choice dimensions, individual change scores were correlated with the cumulative consumption over the 3‐year period. This revealed a negative association between PDG change scores and total alcohol intake (*ρ* = −0.19, CI_95_ = [−0.36, −0.02], *p* = 0.033; compare Figure 4). Consistent with this result, PDG change scores were negatively correlated with the total number of binge‐drinking events (*ρ* = −0.22, CI_95_ = [−0.38, −0.04], *p* = 0.014). This indicates that higher amounts of drinking and more frequent binge drinking were associated with lower increase/higher decrease in risk aversion to gains until age 21 in young men. No significant relations were found for changes in temporal discounting (DD), risk seeking for losses (PDL) or loss aversion (MG) (Table 3).

**TABLE 3:** Spearman correlations between cumulative alcohol consumption and change scores of value‐based decision‐making (VBDM) domains and choice consistency from ages 18 to 21.

VBDM change scores (age_21_–age_18_)^a^	Cumulative alcohol consumption	Spearman correlation		
		*ρ*	CI_95_	*p*‐value
∆ DD log(*k*)	Total intake (kg)	0.07	[−0.11, 0.24]	0.433
	Total binge drinking occasions	0.01	[−0.17, 0.18]	0.951
∆ PDG log(*k*)	Total intake (kg)	−0.19	[−0.36, −0.02]	0.033*
	Total binge drinking occasions	−0.22	[−0.38, −0.04]	0.014*
∆ PDL log(*k*)	Total intake (kg)	−0.06	[−0.23, 0.12]	0.523
	Total binge drinking occasions	−0.02	[−0.19, 0.16]	0.855
∆ MG log(*λ*)	Total intake (kg)	−0.10	[−0.28, 0.08]	0.259
	Total binge drinking occasions	−0.07	[−0.25, 0.11]	0.434
∆ Choice consistency log(*β*)^b^	Total intake (kg)	−0.05	[−0.24, 0.13]	0.573
	Total binge drinking occasions	−0.04	[−0.22, 0.15]	0.700

Abbreviations: CI_95_, 95% confidence interval; DD, delay discounting; MG, mixed gambles (loss aversion); PDG, probability discounting for gains; PDL, probability discounting for losses.

^a^ Change scores were calculated by subtracting log‐transformed values at age 18 from those at age 21.

^b^ Values of choice consistency represent the average of log‐transformed and mean‐centered *β*‐values of DD, PDG, PDL, and MG.

Asterisks refer to the Spearman's correlation coefficient: *p < 0.05.

These results are also in line with cross‐sectional relationships between drinking and VBDM. Whereas at age 18, there were no significant correlations between VBDM dimensions and the average daily consumption the year before baseline, at age 21, risk seeking to gains (lower PDG) was associated with higher daily intake (*ρ* = −0.31, *p* < 0.001), higher AUDIT‐C scores (*ρ* = −0.30, *p* < 0.001) and more frequent binge drinking (*ρ* = −0.27, *p* = 0.002; Table 4), while remaining uncorrelated with the other choice dimensions. Note that AUDIT‐C and binge drinking frequency were not measured at baseline. Regarding choice consistency, change scores were uncorrelated to total alcohol intake and total number of binge drinking events, and there were no cross‐sectional relationships with the past year daily intake, past year binge drinking frequency, or AUDIT‐C.

**TABLE 4:** Cross‐sectional correlations between measures of alcohol use and different domains of value‐based decision making and choice consistency at age 18 and age 21.

VBDM	Alcohol use	Cross‐sectional correlation (Spearman's *ρ*)	
		Age 18	Age 21
DD log(*k*)	Gram/day during past year	−0.007, *p* = 0.936	0.02, *p* = 0.808
	AUDIT‐C		−0.05, *p* = 0.547
	Binge drinking frequency during past year		−0.04, *p* = 0.678
PDG log(*k*)	Gram/day during past year	−0.03, *p* = 0.703	−0.31, *p* < 0.001***
	AUDIT‐C		−0.30, *p* < 0.001***
	Binge drinking frequency during past year		−0.27, *p* = 0.002**
PDL log(*k*)	Gram/day during past year	0.06, *p* = 0.491	−0.03, *p* = 0.770
	AUDIT‐C		0.03, *p* = 0.704
	Binge drinking frequency during past year		−0.03, *p* = 0.768
MG log(*λ*)	Gram/day during past year	0.003, *p* = 0.973	−0.03, *p* = 0.723
	AUDIT‐C		−0.05, *p* = 0.588
	Binge drinking frequency during past year		−0.06, *p* = 0.494
Choice consistency log(*β*)^a^	Gram/day during past year	0.04, *p* = 0.629	0.02, *p* = 0.856
	AUDIT‐C		0.05, *p* = 0.596
	Binge drinking frequency during past year		0.08, *p* = 0.405

Abbreviations: DD, delay discounting; MG, mixed gambles (loss aversion); PDG, probability discounting for gains; PDL, probability discounting for losses.

^a^ Average of log‐transformed and mean‐centered beta values of DD, PDG, PDL, and MG.

Asterisks refer to the Spearman's correlation coefficient: ***p < 0.001, **p < 0.01.

Given the strong correlation between total alcohol intake and the total number of binge drinking events, the question remains whether they uniquely contribute to their association with risk seeking for gains or whether they share a substantial amount of variance. Therefore, partial Spearman correlations were performed to assess the residual strength of the relationship for each drinking measure after accounting for the other. Controlling for total binge drinking frequency, the correlation between total alcohol intake and PDG change scores dropped from *ρ* = −0.19 (*p* = 0.033) to −0.05 (*p* = 0.588), indicating the relationship is largely accounted for by the effect of binge drinking. Controlling for total alcohol intake, the correlation between total binge drinking frequency and PDG change scores decreased from *ρ* = −0.22 (*p* = 0.014) to −0.12 (*p* = 0.189), suggesting that a small though non‐significant negative association remains. These findings may indicate that, although both measures share substantial variance, binge drinking might contribute to altered risk aversion to gains in addition to total alcohol intake.

## DISCUSSION

This longitudinal cohort study investigated the association between alcohol use and development in four dimensions of impulsive choice in early adulthood. Within a community sample of moderate‐drinking men, between the ages of 18–21, we found decreasing preferences for immediate monetary rewards (DD), as well as increasing risk aversion to gains (PDG), that is, a decrease in impulsive choices for immediate and risky rewards. Importantly, and in line with our hypothesis, changes in risk aversion to gains were negatively correlated with cumulative alcohol intake and the total number of binge drinking events over 3 years. This indicates that higher levels of drinking might be associated with a slowed decline in impulsive choice for potential rewards in young men. No changes over time were found in risk seeking to losses (PDL) or loss aversion. Contrary to our hypotheses, cumulative alcohol consumption was not associated with development in DD, risk seeking to losses, or loss aversion.

Our cohort demonstrated decreasing choice impulsivity for delayed and risky rewards during the maturational period of young adulthood. This is consistent with cross‐sectional and longitudinal studies showing a continuous decline in temporal discounting from childhood throughout young adulthood (e.g., de Water et al., 2014; Fröhner et al., 2022; Olson et al., 2007) and quadratic developmental trajectories of risk taking for gains, peaking in mid to late adolescence (e.g., Braams et al., 2015; Cauffman et al., 2010; Rutledge et al., 2016). It also aligns with self‐report measures showing decreases in impulsivity from childhood into adulthood with adolescent peaks of risk taking and sensation seeking (e.g., Spear, 2013; Steinberg et al., 2008). Notably, also stable trajectories of temporal discounting (Audrain‐McGovern et al., 2009; Felton et al., 2020) and risk aversion for gains (de Water et al., 2014; Olson et al., 2007) have been reported. Concerning risk seeking for losses and loss aversion, our findings indicate no substantial changes in young men, consistent with research reporting stable risk taking for losses (Mata et al., 2011; Weller et al., 2011) and loss aversion (Rutledge et al., 2016; Seaman et al., 2018) across age. Notably, also increasing (Gächter et al., 2022) and quadratic (Guttman et al., 2021) age‐patterns of loss aversion and increasing risk taking for losses (Fernandes et al., 2018) were observed in samples spanning much larger age ranges, from young adulthood until middle to old age.

We found that higher alcohol intake and more frequent binge drinking in young men mitigated increasing risk aversion for gains, resulting in higher risk propensity for rewards after 3 years. Consistently, lower risk aversion at age 21, but not age 18, was cross‐sectionally linked to higher alcohol consumption and more frequent binging, while levels of average alcohol use and AUDIT‐C remained stable over time. Although total alcohol intake and binge drinking frequency shared a large variance regarding their relationship toward risk seeking for gains, binge frequency might play an additional role; that is, among individuals with comparable total alcohol exposure, a higher binge drinking frequency might result in augmented effects on risk aversion. One potential mechanism may be that alcohol effects on the brain follow a non‐linear function, and a binge drinking occasion with six drinks might thus be much more harmful than the consumption of single drinks on six occasions (Lees et al., 2019; Ward et al., 2009). However, a higher sample size is necessary to uncover these subtle effects.

Our finding of slowed development in risk aversion for gains among higher drinking individuals aligns with cross‐sectional evidence in adults, showing higher risk taking for gains among heavy drinkers and alcohol‐dependent individuals compared with light drinkers and healthy controls, respectively (Barsky et al., 1997; Bernhardt et al., 2017; Brevers et al., 2014). We previously investigated our study sample to determine how decision‐making domains at baseline predict future alcohol trajectories (Petzold et al., 2023). We found that higher risk seeking for gains at age 18 predicted increasing alcohol consumption and meeting more AUD criteria over the following 6 years. Consistent with this developmental trajectory, the current study shows an association between risk seeking for gains and alcohol use at age 21. Together, these findings suggest a bi‐directional relationship between risk seeking for gains and alcohol use, indicating a scenario where alcohol consumption during young adulthood may be linked to an increased propensity for risky rewards, which further reinforces drinking, thus contributing to a spiral of escalating consumption over time.

According to our hypothesis, we assume that observed relationships between cumulative alcohol use and risk‐taking development are caused by the pharmacological effects of alcohol on the maturing brain. However, according to design, it cannot be ruled out that third factors, such as environmental stressors, might have both influenced how alcohol use and risk taking developed over time. At a pharmacological level, alcohol might have interfered with the maturation of dopaminergic circuits, which continue to develop well into young adulthood (Zucker, 2015). According to neurodevelopmental imbalance models, regions involved in cognitive control, such as prefrontal and parietal areas, and especially their connections to striatal circuits, develop later than areas associated with reward‐driven behavior. These reward‐related regions tend to mature in late adolescence, which may explain adolescent peaks in risk taking and sensation seeking (e.g., Casey et al., 2008; Cyders & Smith, 2008; Somerville & Casey, 2010). Thus, within our study sample, declines in impulsive choice tendencies toward delayed and risky rewards may reflect general growth of impulse control over reward‐driven behavior. This maturational process might have been inhibited in the young men with higher drinking levels, resulting in heightened reward sensitivity and/or reduced control over behavior, such as arousal elicits. Alternatively, or additionally, third factors, such as genetic predispositions or early‐life stress, may underlie both increasing alcohol consumption and slowed development of risk aversion for gains. This consideration is particularly relevant given evidence that certain facets of impulsivity interact with social resources and isolation stress (Heinz, 2017; Heinz et al., 2011).

We found no correlations of alcohol exposure with changes in other dimensions of decision making, that is, temporal discounting, risk seeking to losses, or loss aversion, which aligns with the absence of any cross‐sectional relationships. In cross‐sectional studies of alcohol‐dependent versus healthy adults, differences were evident in other domains of VBDM, such as elevated temporal discounting being repeatedly shown across AUD populations (e.g., Bernhardt et al., 2017; Brevers et al., 2014). For the current study sample, Petzold et al. (2023) reported that in addition to risk seeking for gains, high DD, low loss aversion, and low choice consistency at age 18 each independently predicted increasing alcohol consumption over the following 6 years. The findings of Petzold et al. (2023), along with our results showing no associations between alcohol use and decision‐making development other than risk taking for gains, suggest that elevated DD, lower loss aversion, and lower choice consistency are independent risk factors for escalating alcohol use during youth, rather than being consequences of it. Regarding DD, these findings are in line with other longitudinal studies within young populations (Fröhner et al., 2022).

This contrasts the observed bi‐directional link between alcohol use and risk seeking for gains, indicating that drinking behavior and modifying factors may interact differently across decision‐making domains, rather than universally reinforcing impulsive choice behavior. In light of discussed neurodevelopmental imbalance models, maturing dopaminergic circuits regulating arousal for potential rewards and behavioral control might be more vulnerable during young adulthood. In contrast, other decision‐making domains (related to losses and delayed gratification) may rely on other brain circuits. For example, Guttman et al. (2021) found that thinning of the posterior cingulate cortex mediated age‐related trajectories of loss aversion. Such findings might also explain why domains of decision making probably follow different developmental trajectories, that is, linear decline of temporal discounting versus quadratic pattern of risk seeking to gains (e.g., Braams et al., 2015; de Water et al., 2014). However, further research using functional neuroimaging is essential to uncover the neurofunctional basis of observed differences between altered decision‐making domains. Recognizing such distinctions is critical for understanding the role of impulsivity and risk taking in substance use disorders.

Furthermore, we found no associations between changes in choice consistency and cumulative alcohol use or total binge drinking frequency, which demonstrates that altered development of risk taking seems not to stem from altered consistency of choice behavior, that is, how consistently the difference in option values drives the choice for the subjectively better option. Future research should build on these findings by uncovering how altered neurofunction might underlie the hampered development of risk aversion for gains and identifying possible environmental factors that may modulate this process.

## LIMITATIONS

Most of our participants were highly educated (mean years of formal education = 11.6). A major limitation of the study is the low proportion of high‐risk drinkers in the sample. Only 24% (*N* = 31) had an average daily consumption above 20 g/day during the 3‐year period, and only 4% (*N* = 5) had a daily consumption above 40 g/day. Furthermore, only 19% (*N* = 25) reported an average frequency of at least one binge drinking event per month. Thus, the 6‐year consortium study (LeAD), which provided data for the current analysis, had limited success in recruiting high‐risk drinkers. The reduced variance in drinking behavior might have resulted in an underestimation of effect sizes, thus reducing the probability of detecting potential effects in other VBDM domains. From a pharmacological point of view, it is plausible to assume that higher drinking levels may cause more pronounced effects. Regarding risk seeking for gains, we found effect sizes of about 0.2. Assuming a power of 80% and a significance level of 0.05, the sample size should be at least 194 to detect correlation coefficients of 0.2, indicating that the limited sample size (*N* = 130) might in general be too low to detect associations between alcohol consumption and changes in VBDM domains. Thus, future studies should also include a substantial proportion of high‐risk drinking individuals, probably by using less demanding study designs and aiming for sample sizes of 200 participants or higher.

Furthermore, only men were recruited for our study, following the approved research proposal submitted over a decade ago in 2011. Ample evidence has since demonstrated increasingly comparable drinking levels between sexes, with women also at risk for hazardous alcohol use (e.g., Grant et al., 2017), particularly young women in the age range studied here (e.g., Johnston et al., 2019). Importantly, our results may not generalize to women given sex differences in decision making (e.g., Bos et al., 2013) and sex‐dependent effects of alcohol and related symptoms on adolescent neurocognitive functioning (e.g., Squeglia et al., 2009). Given the modest effect sizes found between alcohol use and changing risk aversion for gains, future studies should investigate larger samples to detect potential sex‐dependent differences in altered decision‐making development.

This study focused on behavioral data only and thus could not clarify whether altered risk behavior corresponds to altered brain function. These alterations may occur within fronto‐striatal circuits regulating sensitivity to rewards and control over reward‐driven behavior, as animal models revealed altered dopamine response in adolescent drinking rats (Schindler et al., 2016). Future research may address such limitations by considering higher sample sizes, including women and individuals with high levels of alcohol use, as well as using paradigms that include functional MRI.

## CONCLUSION

Research on the consequences of drinking on decision making, particularly in youth, is scarce. Most studies focused on predicting hazardous drinking patterns or dependency, often limited to temporal discounting or other impulsivity measures using questionnaires and response inhibition tasks (e.g., Stop‐Signal). This longitudinal study examined the association between cumulative drinking and developmental changes in various decision‐making domains and choice consistency during early adulthood, a period of ongoing brain development. Our findings suggest that alcohol consumption between ages 18 and 21 mitigates increasing risk aversion to gains in young men, resulting in elevated risk taking for gains after 3 years. As risk seeking for gains was previously found to reinforce the development of hazardous drinking patterns, this suggests a spiral of interacting factors that might lead to escalating consumption over time. Future research should focus on the neurofunctional basis of altered changes in risk behavior and its genetic and environmental modifiers, and concentrate on fronto‐striatal circuits that are still maturating in young adults.

## FUNDING INFORMATION

This research was supported by the German Research Foundation (Deutsche Forschungsgemeinschaft), DFG, project numbers: 186318919 (FOR 1617), 178833530 (SFB 940), 402170461 (TRR 265), and 454245598 (IRTG 2773).

## CONFLICT OF INTEREST STATEMENT

The authors declare no conflicts of interest.

## Supporting information


Data S1

## Data Availability

The data that support the findings of this study are available from the corresponding author upon reasonable request.
