# Supplementary material for: Higher alcohol use is associated with subsequent increased risk seeking toward gains: A longitudinal cohort study in young men
Source: Alcohol Clin Exp Res (Hoboken). 2025 Apr 19;49(6):1306–20. doi: 10.1111/acer.70051 (PMC12174501; doi:10.1111/acer.70051)
Supplement: Supplementary file 1 — Data S1 [file ACER-49-1306-s001.docx]

Supplementary Material

| **A** | **DD _log(k)_** | **Figure S1**. Rank-order stability for **A-D)** different domains of value-based decision-making and **E)** choice consistency between age 18 (baseline, BL) and age 21 (36-month follow-up, FU36). Numbers at the top right of each panel refer to the Spearman correlation.  DD = Delay Discounting, PDG = Probability Discounting for Gains, PDL = Probability Discounting for Losses, MG = Mixed Gambles | |
| --- | --- | --- | --- |
|  | |  |  |
| 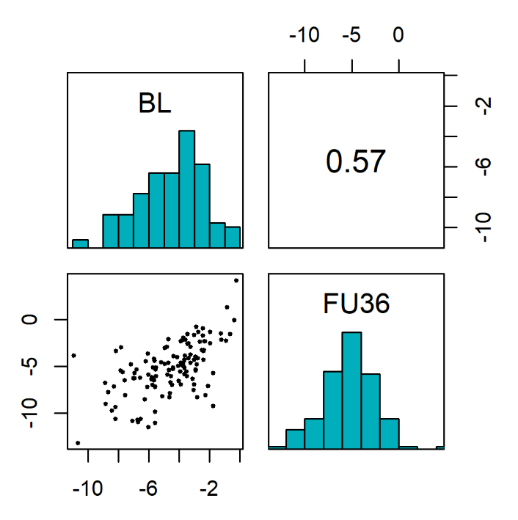 | |  |  |
| **B** | **PDG _log(k)_** | **C** | **PDL _log(k)_** |
|  | | | |
| 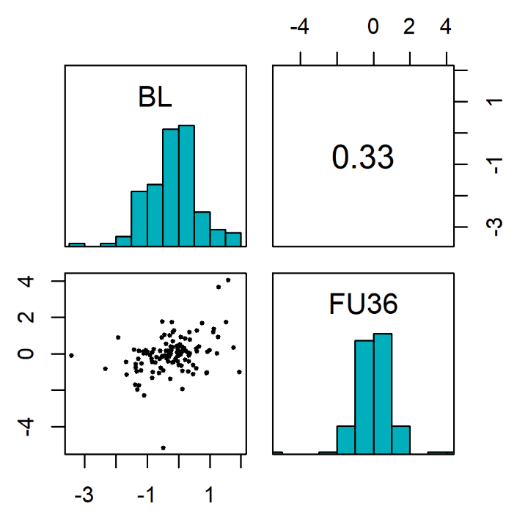 | | 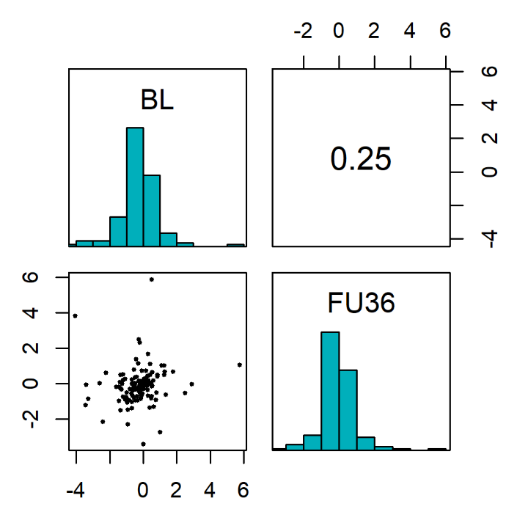 | |
| **D** | **MG _log(λ)_** | E | **Choice consistency _log(β)_** |
|  | | | |
| 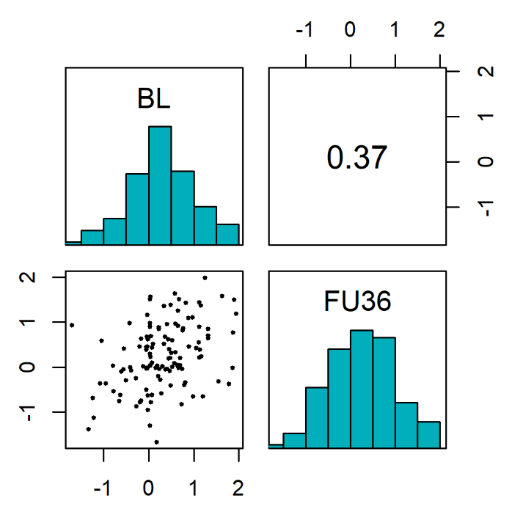 | | 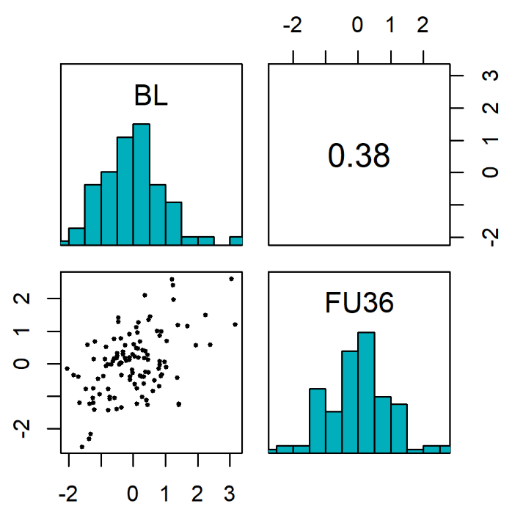 | |

**Impact of drop-out status on baseline levels of VBDM**

From all included individuals at baseline (N = 198), the current study sample only included individuals who had an available estimate of at least one of the four VBDM tasks at the 36-month follow-up (N = 130) to retrieve respective change scores. Thus, dropping out after the baseline (N = 68) might have introduced a bias in task measures. For instance, highly impulsive individuals showing steep temporal discounting might have been more likely to opt out of the study and thus being underrepresented in the sample. To check this, the sample and the drop-out group were compared on baseline values of VBDM. As shown in Fig. S1, Wilcoxon-rank sum tests between the sample and the drop-out group revealed no significant differences in baseline values of any task. Drop-outs might nevertheless have shown different patterns of change over time, different drinking trajectories or different relationships between both.

| 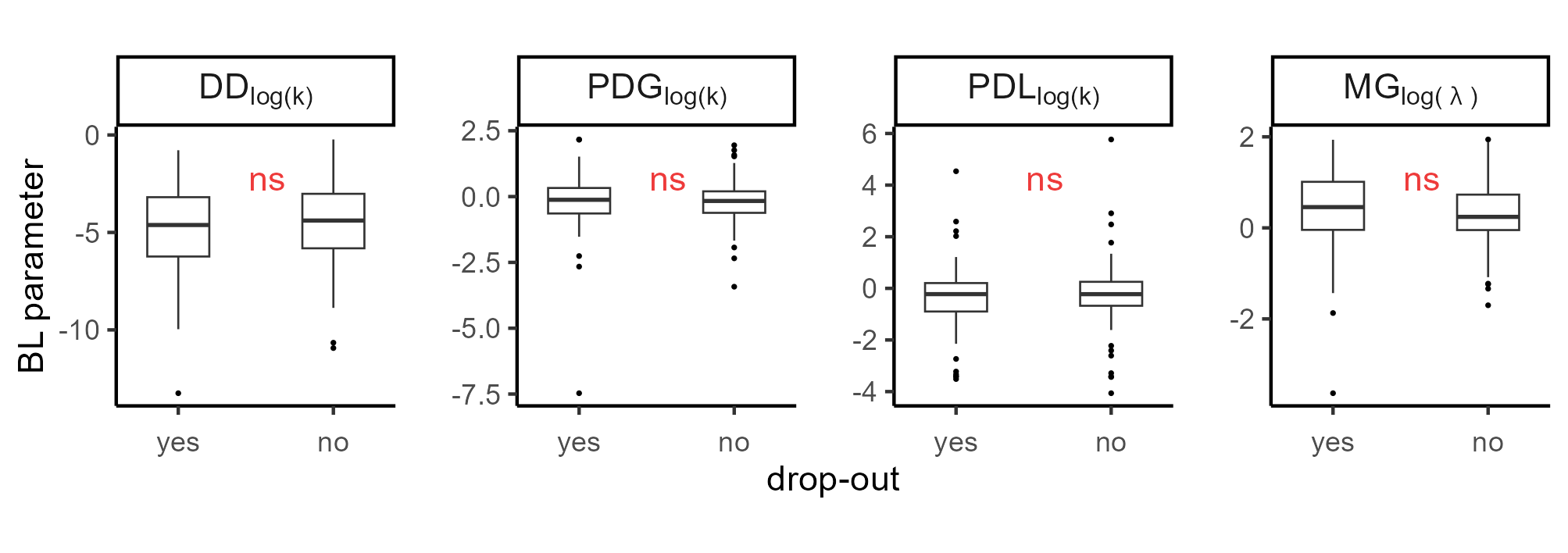 | | | | |
| --- | --- | --- | --- | --- |
|  | *p = 0.761* | *p = 0.967* | *p = 0.722* | *p = 0.266* |

**Figure S2**. Differences in baseline levels of value-base decision-making (VBDM) between the study sample (N = 130) and drop-outs after the baseline (N = 68). p-values represent group differences according to the Wilcoxon-rank sum test.

BL = baseline, DD = Delay Discounting, PDG = Probability Discounting for Gains, PDL = Probability Discounting for Losses, MG = Mixed Gambles

| **A** | **Average daily alcohol intake during past year [gram/day]** | **B** | **AUDIT-C** |
| --- | --- | --- | --- |
| **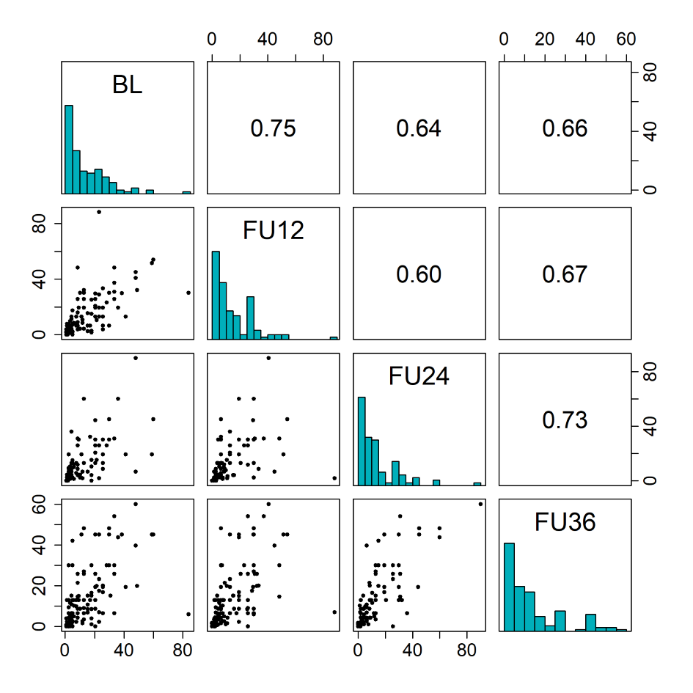** | | 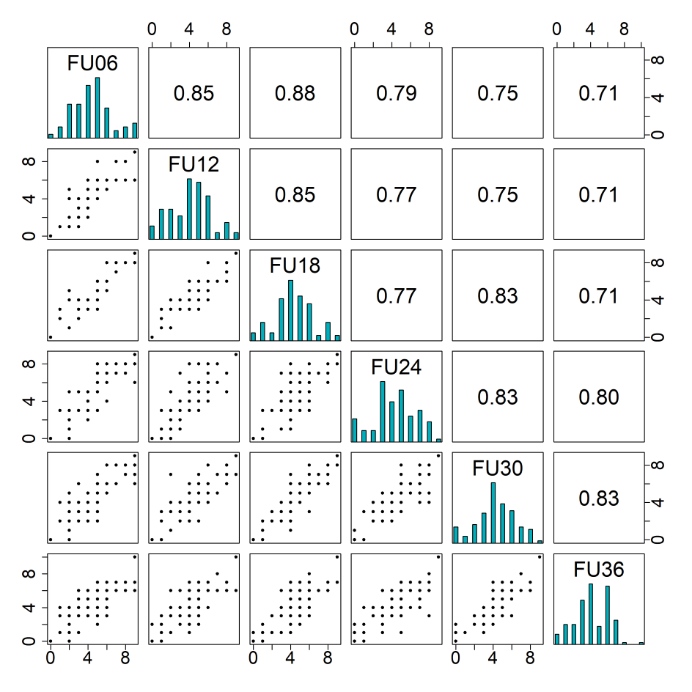 | |
| **C** | **Binge drinking frequency during past year** |  | |
| 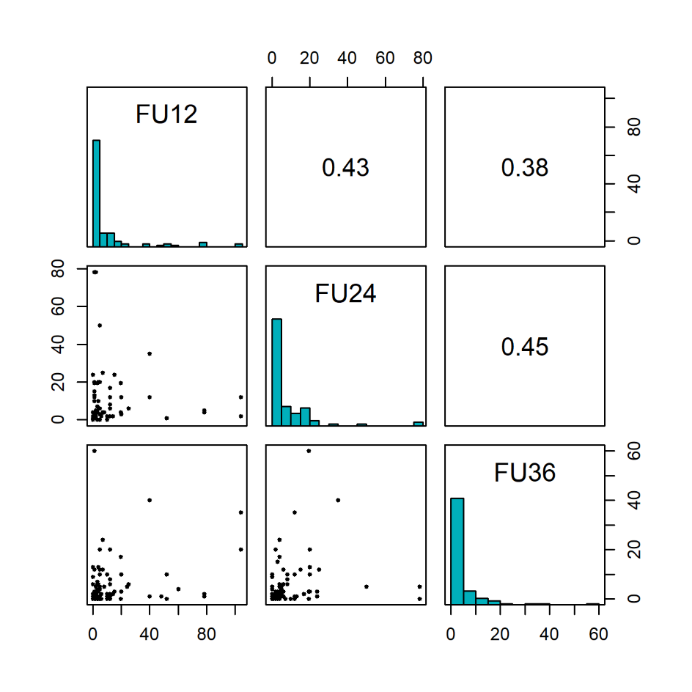 | |  | |

**Figure S3**. Rank-order stability between the repeated measures of alcohol consumption. Numbers at the top right of each panel refer to the Spearman correlation.

BL = baseline, FU = follow-up in months

**Table S1.** Development in cognitive abilities between age 18 and age 21 including working memory, crystallized intelligence and processing speed.

|  | | | | **Wilcoxon signed-rank test (paired)** | | |
| --- | --- | --- | --- | --- | --- | --- |
| **Construct**  Test | **Age** | **Median** | | **W test statistic** | **(pseudo) median**  **[CI_95_]** | **p-value** |
| **Working Memory**  DSST | *18* | 84 | | 5350.5 | 3.50  [1.50, 5.00] | < 0.001 |
|  | *21* | 86 | |  |  |  |
| **Crystallized Intelligence**  MWT-B | *18* | 25 | | 5054 | 1.50  [0.10, 2.50] | < 0.001 |
|  | *21* | 27 | |  |  |  |
| **Processing Speed** |  | | |  |  |  |
| TMT-A | *18* | 20.5 | | 2521.5 | -2.00  [-3.50, -0.50] | 0.002 |
|  | *21* | 20 | |  |  |  |
| TMT-B | *18* | | 46 | 3166.5 | -2.50  [-5.00, 0.00] | 0.058 |
|  | *21* | | 44 |  |  |  |

Differences between both ages were tested using Wilcoxon signed-rank tests for paired samples. DSST = Digit Symbol Substitution Test (Wechsler, 1997), MWT-B = Mehrfach-Wortschatz-Intelligenztest (vocabulary test; Lehrl, 2005), TMT‑A / TMT‑B = Trail Making Test (Reitan, 1979)

CI_95_ = 95% Confidence Interval

**Table S2.** Correlations (Spearman’s Rho) between domains of value-based decision-making (VBDM) at age 18 and age 21, respectively.

| **VBDM domains** | **Age** | **DD log(k)** | **PDG log(k)** | **PDL log(k)** | **MG log(λ)** |
| --- | --- | --- | --- | --- | --- |
| **PDG log(k)** | 18 | -0.03  *p = 0.738* |  |  |  |
|  | 21 | 0.04  *p = 0.651* |  |  |  |
| **PDL log(k)** | 18 | -0.05  *p = 0.590* | 0.04  *p = 0.675* |  |  |
|  | 21 | **-0.25**  ***p = 0.005*** | 0.13  *p = 0.159* |  |  |
| **MG log(λ)** | 18 | 0.004  *p = 0.962* | 0.15  *p = 0.106* | **-0.19**  ***p = 0.036*** |  |
|  | 21 | 0.02  *p = 0.800* | **0.31**  ***p < 0.001*** | **-0.20**  ***p = 0.023*** |  |
| **Choice consistency log(β)^1^** | 18 | -0.16  *p = 0.088* | **-0.20**  ***p = 0.033*** | 0.17  *p = 0.068* | **-0.21,**  ***p = 0.023*** |
|  | 21 | **-0.34**  ***p < 0.001*** | -0.07  *p = 0.443* | -0.04  *p = 0.680* | -0.15  *p = 0.100* |

DD = Delay Discounting, PDG = Probability Discounting for Gains, PDL = Probability Discounting for Losses, MG = Mixed Gambles (loss aversion)

^1^ Values of choice consistency represent the average of log-transformed, mean-centered β-values of DD, PDG, PDL and MG

References

LEHRL, S. 2005. Mehrfachwahl-Wortschatz-Intelligenztest MWT-B 5th Edn. Balingen: Spitta.

REITAN, R. 1979: Trail-Making Test 1979. Arizona: Reitan Neuropsychology Laboratory.

WECHSLER, D. 1997. WAIS-III, Wechsler Adult Intelligence Scale: Administration and Scoring Manual. San Antonio, TX: The Psychological Corporation.
